# Structural and functional changes in the gut microbiota associated to *Clostridium difficile* infection

**DOI:** 10.3389/fmicb.2014.00335

**Published:** 2014-07-04

**Authors:** Ana E. Pérez-Cobas, Alejandro Artacho, Stephan J. Ott, Andrés Moya, María J. Gosalbes, Amparo Latorre

**Affiliations:** ^1^Unidad Mixta de Investigación en Genómica y Salud de la Fundación para el Fomento de la Investigación Sanitaria y Biomédica de la Comunidad Valenciana (FISABIO) y el Instituto Cavanilles de Biodiversidad y Biología Evolutiva de la Universitat de ValènciaValència, Spain; ^2^CIBER en Epidemiología y Salud PúblicaMadrid, Spain; ^3^Institute for Clinical Molecular Biology, Christian-Albrechts-UniversityKiel, Germany; ^4^Department for Internal Medicine, University Hospital Schleswig-HolsteinKiel, Germany

**Keywords:** Gut microbiota, bacterial composition, metabolic functions, *C. difficile* infection, colonization resistance

## Abstract

Antibiotic therapy is a causative agent of severe disturbances in microbial communities. In healthy individuals, the gut microbiota prevents infection by harmful microorganisms through direct inhibition (releasing antimicrobial compounds), competition, or stimulation of the host's immune defenses. However, widespread antibiotic use has resulted in short- and long-term shifts in the gut microbiota structure, leading to a loss in colonization resistance in some cases. Consequently, some patients develop *Clostridium difficile* infection (CDI) after taking an antibiotic (AB) and, at present, this opportunistic pathogen is one of the main causes of antibiotic-associated diarrhea in hospitalized patients. Here, we analyze the composition and functional differences in the gut microbiota of *C. difficile* infected (CDI) vs. non-infected patients, both patient groups having been treated with AB therapy. To do so we used 16S rRNA gene and metagenomic 454-based pyrosequencing approaches. Samples were taken before, during and after AB treatment and were checked for the presence of the pathogen. We performed different analyses and comparisons between infected (CD+) vs. non-infected (CD−) samples, allowing proposing putative candidate taxa and functions that might protect against *C. difficile* colonization. Most of these potentially protective taxa belonged to the Firmicutes phylum, mainly to the order Clostridiales, while some candidate protective functions were related to aromatic amino acid biosynthesis and stress response mechanisms. We also found that CDI patients showed, in general, lower diversity and richness than non-infected, as well as an overrepresentation of members of the families Bacteroidaceae, Enterococcaceae, Lactobacillaceae and Clostridium clusters XI and XIVa. Regarding metabolic functions, we detected higher abundance of genes involved in the transport and binding of carbohydrates, ions, and others compounds as a response to an antibiotic environment.

## Introduction

The human intestinal microbiota is involved in many host functions, such as food processing, regulating intestinal epithelium growth, immune system development, synthesis of essential vitamins, or protection against pathogens (Hooper et al., 2002; Guarner and Malagelada, 2003; Hattori and Taylor, 2009; Leser and Mølbak, 2009; Montalto et al., 2009). Because of its role in human health, imbalances in the gut microbiota have been associated to pathologies such as inflammatory bowel disease, diabetes, obesity, or Crohn's disease (Kang et al., 2010; Sekirov et al., 2010; Morgan et al., 2012; Shanahan, 2013). Antibiotic (AB) therapy has been crucial to treat bacterial infections for over half a century, but it strongly disturbs the gut commensal bacteria and, consequently, the beneficial functions they perform (Jernberg et al., 2010; Willing et al., 2011; Pérez-Cobas et al., 2013a). In fact, AB usage has been associated to short and long-term changes in the intestinal microbiota, reducing colonization resistance to opportunistic pathogens such as *Clostridium difficile* (Vollaard and Clasener, 1994; Bartlett, 2002; Jernberg et al., 2010; Reeves et al., 2011; Britton and Young, 2012). *C. difficile* is an anaerobic, spore-forming, Gram-positive toxin-producing bacterium, which is the most common cause of nosocomial diarrhea, and broad spectrum ABs constitute one of the primary risk factors for infection by this pathogen (Hookman and Barkin, 2009; Cohen et al., 2010). Under normal conditions, the human gut microbiota is able to prevent pathogen invasion through general mechanisms such as direct inhibition (by releasing inhibitory compounds, bacteriocins), nutrient depletion (consuming growth-limiting nutrients) or stimulating host immune defenses (reviewed in Stecher and Hardt, 2011). The exact mechanism by which the microbiota protects against *C. difficile* infection (CDI), preventing its growth and virulence, is still unknown. In this regard, direct antagonism was found *in vitro* since *C. difficile* is a target of bacteriocin produced by an intestinal strain of *Bacillus thuringensis* (Britton and Young, 2012). Since the gut microbiota participates actively in the fermentation of diet carbohydrates, amino acid and lipid metabolism and protein digestion, Theriot et al. used a metabolic model of CDI in mice and found that ABs affect all these functions, leading to a disturbed microbiota functional state that favors *C. difficile* germination and growth (Theriot et al., 2014). Moreover, gut microorganisms participate in bile acid transformation, which otherwise stimulate *C. difficile* spore germination and growth (Britton and Young, 2012). Thus, the loss of key taxa which play these roles can trigger a structural and functional imbalance, allowing colonization by this opportunistic pathogen.

In recent years, high-throughput molecular techniques, such as 16S rRNA gene sequence analyses (taxonomic composition of microbial communities), metagenomics (genetic potential of microbial communities) and other meta-“omics” (metatranscriptomics, metaproteomics, metabolomics) have extended our knowledge of intestinal microbiota diversity and functions (Gill et al., 2006; Kurokawa et al., 2007; Zoetendal et al., 2008; Tap et al., 2009; Gosalbes et al., 2011; Pérez-Cobas et al., 2013a,b). Some of these approaches have recently been used to address the effects of ABs in the gut ecosystem (Dethlefsen et al., 2008; Antonopoulos et al., 2009; Dethlefsen and Relman, 2010; Jakobsson et al., 2010; Antunes et al., 2011; Pérez-Cobas et al., 2013b) showing that ABs considerably alter the gut microbial ecology and the host-microbiota interactions (Pérez-Cobas et al., 2013a). The response of the microbiota to ABs is related to properties of the agent, such as the antimicrobial effect, mode of action, dosage and duration of treatment, or route of administration (Jernberg et al., 2010; Pérez-Cobas et al., 2013b). In addition, biological factors of the host-microbial ecosystem itself such as taxonomic and functional composition, resistance gene reservoir, or host immune homeostasis also contribute to the microbial community shifts associated to AB therapy (Jernberg et al., 2010; Willing et al., 2011; Relman, 2012). To date, few studies have aimed to ascertain whether specific changes in the microbiota composition due to AB therapy lead to CDI. Past surveys have shown that diversity of the intestinal microbiota is significantly reduced in patients prior and/or during CDI, as well as important structural changes associated to infection (Antharam et al., 2013; Vincent et al., 2013).

The main goal of the present follow-up study is to gain insights into the development of CDI and its relation to an altered human gut microbiota. We have used 16S rRNA gene and metagenomic approaches to characterize the structure and functions of the intestinal microbiota before, during and after broad spectrum AB therapy in patients who developed CDI. In two previous studies we explored the effect of broad spectrum ABs on human gut microbiota composition and function in patients that did not develop CDI at any time (Pérez-Cobas et al., 2013a,b). Comparative analyses of these two groups of patients identified bacterial taxa and metabolic functions associated to an infection status, as well as specific taxa and functions that could protect against the *C. difficile*, and thus contribute to colonization resistance of the human gut microbiota.

## Materials and methods

### Sample collection

Three patients under AB therapy at the Department of Internal Medicine of the University Hospital Schleswig-Holstein, Kiel, Germany were recruited for the study due to the fact that they were positive for *C. difficile* at some time points of the treatment. The patients were older than 65 years, no antibiotic therapy was administered to them in the previous 6 months to their hospital admission. The diagnosis at the entrance to the hospital were ischaemic colitis, sigmoid diverticulitis and infection of unknown origin for patients referred as F, G, and H, respectively. The patients stayed in the hospital during the AB therapy. Written, informed consent was obtained from all the subjects.

Fecal samples were collected, before, during and after AB treatment, from the three patients in sterile tubes and stored at −80°C until processing all sample together. Fecal samples were screened by multiplex PCR for *C. difficile* toxin genes, *tcdA* and *tcdB*, and triose phosphate isomerase gene (*tpi*), considering *C. difficile* positive those samples that resulted positive for the three examined genes (referred as CD+, whereas CD− is used for the rest of samples). Patients F and H were found positive after 16 and 35 days after AB treatment, respectively, whereas patient G was found positive on entering hospital (Table 1). The three patients presented diarrhea during AB theraphy.

**Table 1:** **Description of the patients involved in the study**.

**Patient**	**Antibiotic**	**Sampling date**	**Samples code**	**Study**	**CD (+/−)**
A	Moxifloxacin	Day0—before AB	A_before	Pérez-Cobas et al., 2013b	−
		Day3—during AB	A3_D		−
		Day6—during AB	A6_D		−
		Day10—during AB	A10_D		−
		Day13—during AB	A13_D		−
B	Clindamycin	Day0—before AB	B_before	Pérez-Cobas et al., 2013b	−
		Day2—during AB	B2_D		−
		Day5—during AB	B5_D		−
		Day6—during AB	B6_D		−
C	Cefazolin/Ampicillin/Sulbactam	Day0—before AB	C_before	Pérez-Cobas et al., 2013b	−
		Day3—during AB	C3_D		−
		Day6—during AB	C6_D		−
		Day10—during AB	C10_D		−
D	Amoxicillin	Day0—before AB	D_before	Pérez-Cobas et al., 2013b	−
		Day3—during AB	D3_D		−
E	Ampicillin/Sulbactam/Cefazolin	Day0—before AB	E_before	Pérez-Cobas et al., 2013a	−
		Day3—during AB	E3_D		−
		Day6—during AB	E6_D		−
		Day11—during AB	E11_D		−
		Day14—during AB	E14_D		−
F	Amoxicillin/Ciprofloxacin/Clarithromycin	Day0—before AB	F_before	Present study	−
		Day16—during AB	F16_D		+
		3 days after AB	F_after		+
G	Ciprofloxacin	Day0—before AB	G_before	Present study	+
		Day4—during AB	G4_D		+
		4 days after AB	G_after		+
H	Vancomycin/Ampicillin/Sulbactam	Day0—before AB	H_before	Present study	−
		Day7—during AB	H7_D		−
		Day14—during AB	H14_D		−
		Day20—during AB	H20_D		−
		Day35—during AB	H35_D		+
		Day38—during AB	H38_D		+
		26 days after AB	H_after		−

CD (+/−), positive and negative detection for C. difficile. AB, antibiotic.

In two previous studies we evaluated the effect of broad-spectrum antibiotics on five patients (A, B, C, D, E) through similar approaches of those presented in this work (16S rRNA gene and metagenomics) as part of the same research survey (Pérez-Cobas et al., 2013a,b) that was approved by the Ethical Committee of the University Hospital Schleswig-Holstein. None of these patients developed CDI (they were negative for the multiplex PCR for *C. difficile tcdA, tcdB*, and *tpi* genes), or presented diarrhea. The main features and therapy of all patients (A, B, C, D, E, F, G and H) are shown in Table 1. Only the time-points used in this study are shown for the patients A, B, C, D, and E (all CD− samples) of the previous studies.

### DNA extraction and sequencing process

The fecal samples were resuspended in sterile PBS [containing, per liter, 8 g of NaCl, 0.2 g of KCl, 1.44 g of Na_2_HPO_4_, and 0.24 g of KH_2_PO_4_ (pH 7.2)] and centrifuged at 1250 g and 4°C for 2 min to remove fecal debris. The supernatants were centrifuged at maximum speed at 4°C for 5 min to pellet the cells. DNA was extracted with the QIAamp® DNA Stool Kit (Quiagen) following the manufacturer's instructions. Total DNA integrity was checked by running a standard agarose gel electrophoresis and the concentration was quantified with the QuantiT PicoGreen dsDNA Assay Kit (Invitrogen). For each sample, except of F_after from which there was no enough amount of DNA, the total DNA (metagenome) was directly pyrosequenced with a Roche GS FLX sequencer and Titanium chemistry in the Center for Public Health Research (FISABIO-Salud Pública) (Valencia, Spain). Thus, a total of 12 metagenomes were analyzed. We obtained a mean of 78,976 reads per sample with an average length of 374 bp.

### 16S rRNA gene amplification

A region of the 16S rRNA gene (V1, V2, and V3) was amplified by polymerase chain reaction (PCR) for each sample. The primers were the universal E8F (5′-AGAGTTTGATCMTGGCTCAG-3′) with adaptor A and 530R (5′-CCGCGGCKGCTGGCAC-3′) with adaptor B using the sample-specific Multiplex Identifier (MID) for pyrosequencing according to 454 standard protocols. For each sample a 50 μl PCR mix was prepared, containing 5 μl of Buffer Taq (10X) with 20 mM MgCl2, 2 μl of dNTPs (10 mM), 1 μl of each primer (10 mM), 0.4 μl of Taq Fast start polymerase (5 u/μl), 39.6 μl of nuclease-free water and 1 μl of DNA template. PCR conditions were: 95°C for 2 min followed by 25 cycles of 95°C for 30 s, 52°C for 1 min and 72°C for 1 min and a final extension step at 72°C for 10 min. The amplification products were checked by electrophoresis in agarose gel (1.4%). PCR products were purified using NucleoFast® 96 PCR Clean-Up Kit (Macherey-Nagel) and quantified with the QuantiT PicoGreen dsDNA Assay Kit (Invitrogen). The pooled PCR products were directly pyrosequenced in the same way as the total DNA (described above). We obtained an average of 5394 reads per sample.

### Analysis of the 16S rRNA gene reads

We used the Ribosomal Database Project (RDP) pipeline (Cole et al., 2009) to trim off the MID and primers and also to filter sequences by quality. Sequences with a phred quality score below 20 (Q20) and short length (<250 bp) were discarded. The denoising of the sequences was performed with the usearch program in the QIIME pipeline (Caporaso et al., 2010). Then, the pyrosequencing chimeras were discarded using the uchime filtering also in the QIIME pipeline. After, OTUs were calculated at 97% of sequence similarity by clustering with the usearch program in the QIIME pipeline. The taxonomic assignment of the amplicons was based on the database of RDP. We included only annotations obtained with a confidence level (bootstrap cut-off) greater than 0.8, leaving the assignment at the last-well identified level and the consecutive levels as unclassified (uc).

### Biodiversity and ecological analysis

To analyze the microbial community structure at OTU level (97%) we calculated two diversity parameters: number of OTUs and Shannon index (Shannon, 1948) and two richness estimators: Chao1 (Chao, 1984) and abundance-based coverage (ACE) (Chao et al., 2000). These estimators are implemented in Vegan package (Oksanen et al., 2011) under R software (http://cran.r-project.org) (R Development Core Team, 2011). To statistically compare the mean ranks of the biodiversity measures between groups, we used the Wilcoxon signed-rank test implemented in the R software.

We also performed a clustering based on OTU composition to study the similarity between samples using the pvclust library (Suzuki and Shimodaira, 2006) in the R software. This analysis assesses the uncertainty in hierarchical clusters using bootstrap resampling techniques. We used the approximately unbiased (AU) *p*-value with 10,000 bootstraps to estimate the probability of each cluster. This AU *p*-value indicates how strong the cluster is supported by data.

### Functional classification of metagenomes

We used the 454 Replicate Filter Program (Gómez-Alvarez et al., 2009) to eliminate artifact replicate reads of pyrosequencing following the parameters: sequence identity cutoff = 1; length difference requirement = 0; number of beginning base pairs to check = 10. Reads were compared against the human genome database using BLASTN (Altschul et al., 1990) with an *e*-value of 10^−10^ to eliminate possible contamination with human sequences. To identify the sequences encoding the ribosomal 16S rRNA and 23S rRNA genes we compared the dataset against the Small Subunit rRNA Reference Database (SSUrdb) and the Large Subunit rRNA Reference Database (LSUrdb) described in Urich et al. (2008) using BLASTN with an *e*-value of 10^−16^ and 10^−4^ respectively. After removing the ribosomal genes, the remaining reads were compared to the NCBI-nr protein database using BLASTX (Altschul et al., 1990) to identify the protein-coding genes, and then we performed an Open Reading Frames (ORFs) search with the Fraggenscan program from the metagenomic analysis web server (WebMGA) (Wu et al., 2011). The predicted ORFs were functionally annotated by HMMER 3.0 (Durbin et al., 1998) against the TIGRFAM database (Haft et al., 2003) using default parameters.

### Statistical analysis

Canonical correspondence (CCA) and detrended correspondence (DCA) analyses were performed to explore the relationship between different groups of samples and with *C. difficile* infection as a variable that could explain the variability pattern. To statistically assess the effect of that variable in explaining the bacterial composition differences, a multivariate ANOVA based on dissimilarity tests (Adonis) was applied, implemented in the Vegan package under the R software. These approaches were applied to two different levels: the taxonomy based on the 16S rRNA gene, and the biological functions based on the TIGRFAMs annotations. We used the ShotgunFunctionalizeR package (Kristiansson et al., 2009) in the R software to statistically compare samples at diversity and functional levels. The differences in composition between samples were addressed comparing groups of multiple samples with the function “testGeneFamilies.dircomp.” On the other hand, we applied the “testGeneCategories.dircomp” function to compare the distribution of functional categories between groups of samples. It compares each gene family from a higher functional category to decide whether the global category is statistically significant among two groups of samples. All tests were based on Poisson models.

### Searching for putative candidate taxa and metabolic functions to protect against CDI

We also used the “testGeneCategories.dircomp” test to identify taxa and metabolic functions that could play a protective role against *C. difficile* colonization. Specifically, we performed three comparisons between groups of samples to identify taxa and functions that were significantly over-represented in CD− compared to CD+ samples. The taxa and functions resulting from the different comparisons were intersected to define the candidate protective group. For this purpose, we performed the following comparisons:

Comparison 1.Since patients F and H were negative to the pathogen before treatment but positive during therapy, we compared the CD− samples before AB (F_before and H_before) against the CD positive samples (CD+) during AB (F_16D, H35_D and H38_D) (Table 1). We aimed to identify taxa and functions that significantly decreased (*p*-value < 0.1) due to treatment, presumably allowing *C. difficile* overgrowth.

Comparison 2. Since patients A, B, C, D and E did not develop CDI, we performed a comparison of the samples before AB treatment against their samples during treatment (Table 1). We aimed to identify taxa and functions that significantly increased (*p*-value < 0.1) due to therapy or that changed less drastically than those in Comparison 1, since their presence could play a role in preventing *C. difficile* infection.

Comparison 3. Since patient H was negative for the pathogen 26 days after AB, we carried out a comparison of the CD+ samples of patient H (H35_D and H38_D) against the CD− sample taken after AB (H_after) (Table 1). We aimed to identify taxa and functions whose significant increase (*p*-value < 0.05) could be incompatible with pathogen overgrowth as this was not detected.

Finally, we intersected all these results to obtain a group of candidate taxa and functions that could participate in *C. difficile* colonization resistance.

### Co-occurrence bayesian networks of candidates (taxa and metabolic functions) in CD− samples

To find positive correlations between candidate protective taxa or functions found in the previous analyses and other taxa and functions obtained for samples from patients A, B, C, D and E during AB treatment (all CD− samples), we performed Bayesian networks based on their relative abundance. The Bayesian networks were inferred using the bnlearn package (Scutari, 2010) in the R software. The optimal network inference was constrained so that only those interactions exhibiting a Spearman correlation *p*-value below 0.01 were included in the network. Correlations and *p-values* were computed using the Spearman method implemented in R software.

### Data accession number

All sequences have been entered in the European Bioinformatics Institute database, under accession numbers: ERP002192 (patients A, B, C, and D), ERP001506 (patient E) and PRJEB5771 (patients F, G, and H).

## Results

### Microbial diversity and bacterial composition in patients developing CDI

**Figure 1:**
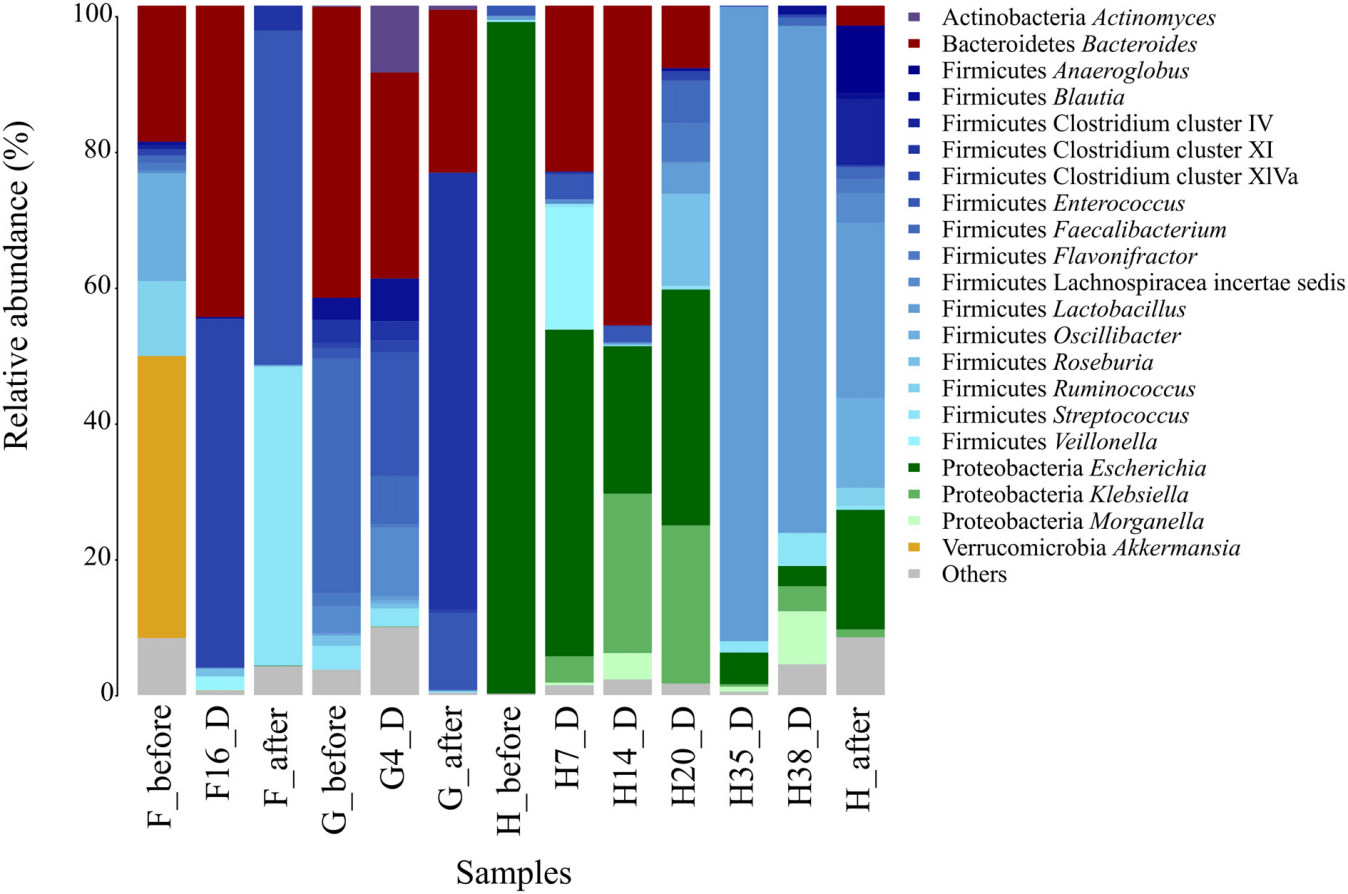
**Fecal microbiota composition in CDI patients (F, G, and H).** The composition of each sample is based on the RDP taxonomic assignment of the 16S rDNA sequences. The phylum and the genus level are shown for the most abundant bacterial groups (>5%).

Analysis of the gut microbiota of the three CDI patients (F, G, and H) showed large variations in bacterial composition during therapy (Figure 1).

Before AB treatment, the bacterial composition of patient F was dominated by the *Akkermansia* genus (30.6%) belonging to the family Verrucomicrobiaceae. Other bacterial families were also abundant such as Ruminococcaceae (20.8%), Oscillibacteriaceae (*Oscillibacter*, 11.7%) and Bacteroidaceae (*Bacteroides*, 14.8%). When *C. difficile* was detected, at day 16 of AB treatment, all these taxa were almost absent in the community except *Bacteroides*, which had increased to 41.9%, becoming a predominant genus of the gut ecosystem. The Clostridium cluster XlVa increased dramatically (from 0.7% before AB course to 46.8% at day 16), being the most abundant group at this time point. After treatment, the abundance of the main taxa of the microbial community changed again, the predominant being Enterococcaceae *(Enterococcus*, 48.3%), Streptococcaceae (*Streptococcus*, 43.2%), Staphylococcaceae (*Staphylococcus*, 4.1%) and Clostridium cluster XI (3.5%).

Patient G was found positive to *C. difficile* detection before, during, and after AB treatment, showing the most similar bacterial composition at the three time points, though there are some remarkable differences. The initial composition (G_before) consisted mostly of Bacteroidaceae (*Bacteroides*, 36.7%) and Ruminococcaceae (*Faecalibacterium*, 29.6%). During AB (G4_D), although *Bacteroides* decreased in abundance to 25%, it remained the most abundant genus, while *Faecalibacterium* (5.9%) decreased radically. However, *Enterococcus* increased during AB (from 1.3 to 14.9%). After therapy (G_after), Clostridium cluster XI became the predominant group (62.4%) whereas *Streptococcus* genus decreased progressively at each time point (3, 2.2, and 0.2%, respectively).

Patient H had a very unusual gut microbiota before AB treatment, being dominated (85.7%) by Enterobacteriaceae family, mainly *Escherichia* genus, but its abundance decreased dramatically reaching the lowest values at days 35 and 38 of the broad-spectrum AB treatment (4.2 and 2.8%, respectively), when *C. difficile* was detected. During days 7 and 14 of AB treatment the genus *Bacteroides* showed the higher abundance values (20.4 and 34.8%); however this taxon decreased on day 20, becoming undetectable by days 35 and 38. *Streptococcus* genus increased slightly in the two CD+ samples (1.5 and 4.5%, respectively). The most striking shift occurred in the Lactobacillaceae family (*Lactobacillus* genus), whose frequency increased from less than 1% at the beginning of treatment to 83.3 and 70% on days 35 and 38 of the AB course, and was reduced to 15.5% after AB. We performed a statistical comparison to evaluate the differences in bacterial composition between the samples immediately prior to *C. difficile* detection (H14_D, H20_D) and in the CD+ samples (H35_D, H38_D). (Table S2). The main significantly overrepresented taxa were *Lactobacillus, Streptococcus, Proteus, Sutterella* and the uc_Lactobacillaceae, while the Clostridium cluster XlVa, *Enterococcus, Bacteroides, Escherichia, Klebsiella*, and *Roseburia* were the least abundant taxonomic groups.

The three individuals exhibited great fluctuations in the number of observed OTUs, as well as in the diversity parameters analyzed (Table S1). The diversity (based on Shannon, Chao 1 and ACE estimators) of patient F was reduced in the CD+ samples, being minimal after the therapy. The microbial diversity of patient G also reached the lowest values after treatment. The decreased diversity after the course in these two patients could be due to both, the AB and CDI effects. However, the patient H, which was recovered of the infection after the therapy, presented the lowest diversity parameters before the AB that could be due to the massive presence of members of the Enterobacteriaceae family detected in this sample and also during CDI (Figure 1).

**Figure 2:**
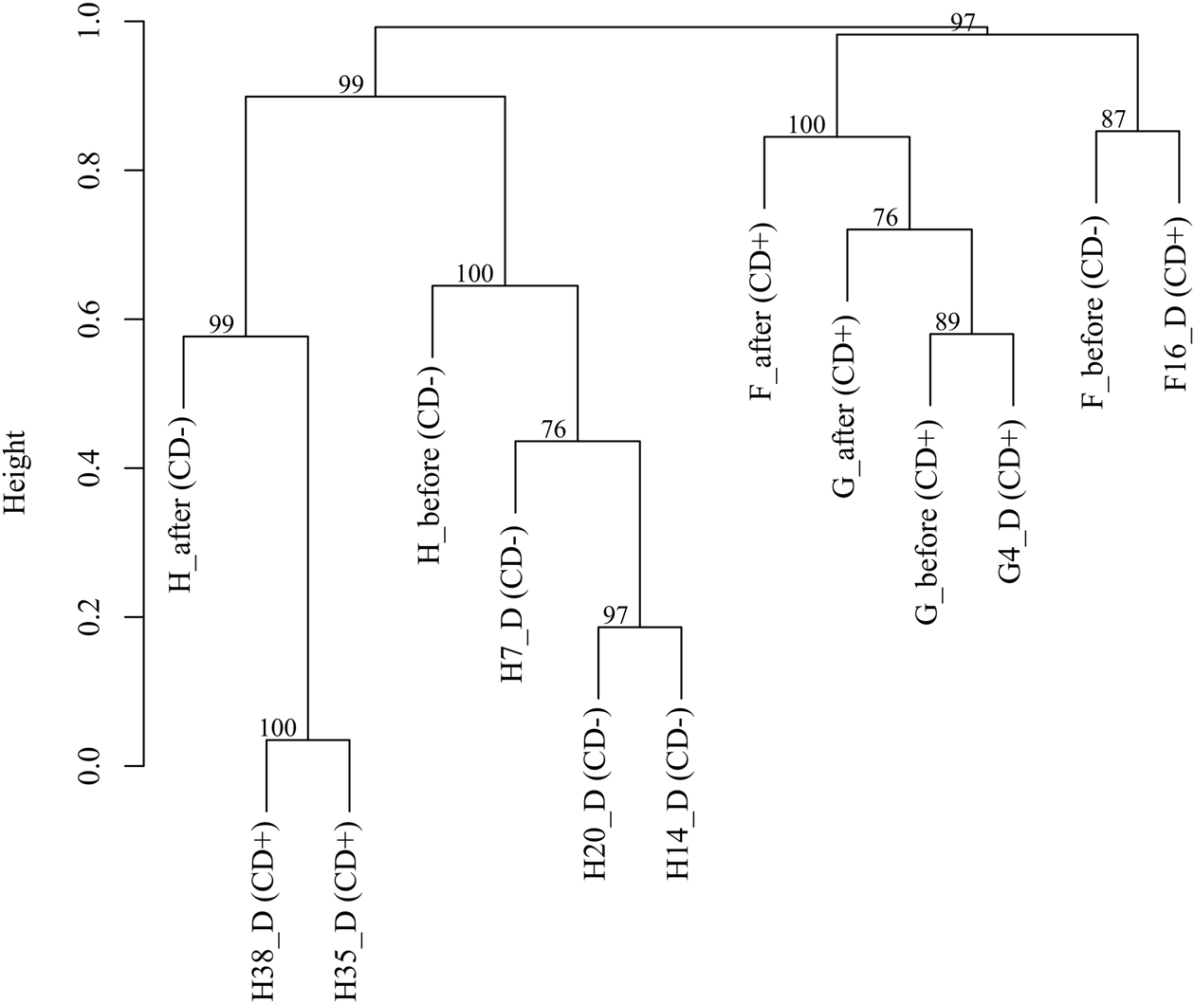
**Cluster analysis based on OTUs (97%) of CDI patients (F, G, and H).** The approximate unbiased (AU) *p*-values are shown.

Finally, we performed a cluster analysis to find similarities in microbiota composition between samples at OTU level (97%) (Figure 2). The three samples corresponding to patient G (G_before, G4_D and G_after) were clustered with F_after, being all CD+ samples. This cluster was closer to the others two samples of patient F (F_before and F16_D). Patient H samples formed two clear groups. One of the clusters included the prior infection samples (before AB and 7, 14 and 20 days during AB) whereas the CD+ samples (days 35 and 38), which are the most similar samples, grouped in a second cluster with the sample after treatment (H_after). The clustering shows that both the individual and the *C. difficile* presence contributed to explain the similarity pattern of the samples.

### Differences in microbial structure between *c. difficile*-infected and non-infected patients

In previous studies we analyzed changes in bacterial composition in AB-treated patients that did not develop *C. difficile* infection (A, B, C, D and E), and thus all samples were CD−. To search for differences in microbiota composition possibly related to infection, we compared the 15 time points during the AB therapy of these CD− patients with samples from patients that were positive for *C. difficile* detection (CD+) (F16_D, F_after, G_before, G4_D, G_after, H35_D and H38_D) (Table 1).

First, we compared the Shannon index distributions between CD+ and CD− samples (Figure S1). We found a lower diversity for CD+, with an average of 3.1 ± 1.0 compared to CD− samples with 3.9 ± 0.8, respectively. The richness estimator, Chao1, showed great variations for both groups; even so, the means were also lower in the CD+ populations with values of 210 ± 132 vs. 287 ± 157 in CD− individuals. The Wilcoxon signed-rank test was performed to compare the diversity measures between both groups, and was not significant for the Shannon index (*p* = 0.14) and the Chao1 estimator (*p* = 0.33). The gut microbiota of CD+ samples seems to be more heterogeneous and less rich than the CD− samples corresponding to patients that did not develop CDI, but a larger number of samples would be required to support this observation.

**Figure 3:**
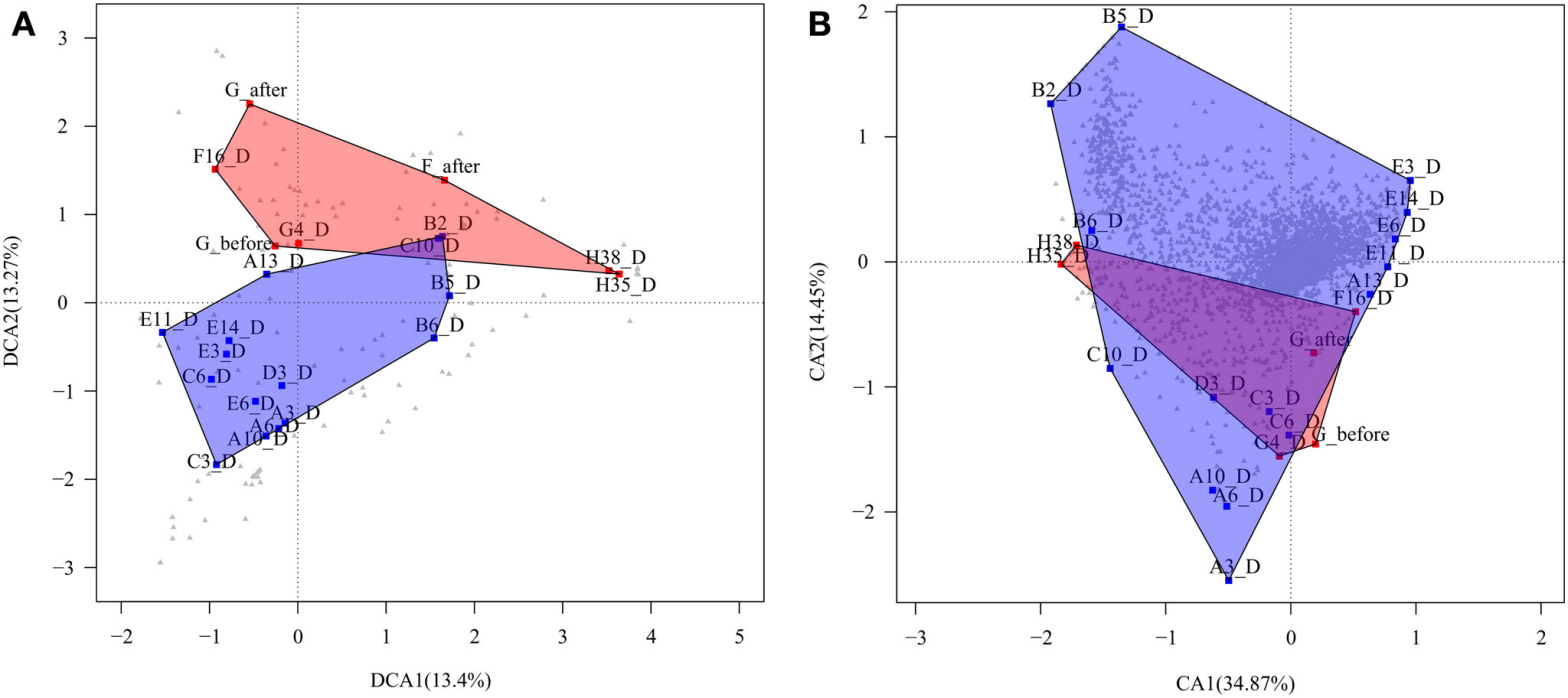
**Correspondence analyses. (A)** Detrended correspondence analysis (DCA) based on taxa abundance and composition of CD+ samples of patients (F, G, and H) (red square) and CD− samples of patients (A, B, C, D, and E), during treatment (blue square). Gray triangle indicates taxa distribution. **(B)** Correspondence analysis (CA) based on the functional profile (TIGRFAMs) of the same samples. Gray triangle indicates functions.

Second, we performed a detrended correspondence analysis (DCA) to explore the variations in bacterial composition between the same CD+ and CD− samples tested above (Figure 3A). The two axes explained 26. 7% of the total variance, and there was large variability in the microbiota of both groups. Despite this variability, two clusters can be distinguished with minimal overlapping. We applied the Adonis test to evaluate whether developing *C. difficile* infection is a factor that influences the microbiota structure. The factor proved to be significant with a *p*-value of 0.005.Thus, although the CD+ samples do not form a well-defined cluster, they share some features in their microbiota composition that differ from CD− samples.

Finally, we performed a statistical test to find those taxa that explained the differences in composition between CD− and CD+ groups. In CD+ samples there was significant over-representation of the genera *Lactobacillus, Bacteroides, Enterococcus, Faecalibacterium*, the family Lachnospiraceae incertae sedis, and the Clostridium clusters XIVa and XI, the latter included *C. difficile*. However, commensal members of the intestinal community, such as *Roseburia, Coprococcus, Blautia, or Subdoligranulum* genera and the families Erysipelotrichaceae and Ruminococcaceae were underrepresented (Table 2).

**Table 2:** **Differential taxa abundance between CD− (during time points of A–E patients) and CD+ (F16_D, F_after, G_before, G4_D, G_after, H35_D and H38_D) samples**.

**Taxa**	**Abundance in CD+ samples**	***P*-value**
Clostridium cluster XlVa	Increase	0
Clostridium cluster XI	Increase	0
*Lactobacillus*	Increase	0
*Bacteroides*	Increase	0
Lachnospiracea incertae sedis	Increase	5.65E-137
*Faecalibacterium*	Increase	2.30E-121
*Enterococcus*	Increase	7.06E-120
uc_Lachnospiraceae	Decrease	0
*Blautia*	Decrease	0
uc_Ruminococcaceae	Decrease	0
uc_Enterobacteriaceae	Decrease	0
*Roseburia*	Decrease	0
*Parabacteroides*	Decrease	0
*Subdoligranulum*	Decrease	0
*Oscillibacter*	Decrease	0
*Coprococcus*	Decrease	2.01E-84
*Alistipes*	Decrease	1.78E-46
uc_Erysipelotrichaceae	Decrease	8.51E-54
*Butyricicoccus*	Decrease	1.18E-48
*Lactococcus*	Decrease	1.62E-46
*Streptococcus*	Decrease	0.01

### Candidate taxa involved in *c. difficile* colonization resistance

In order to obtain a subset of candidate bacteria that could be involved in *C. difficile* colonization resistance, we performed statistical comparisons between different groups of samples (see Materials and Methods for the three specific comparisons). The three comparative analyses gave a number of statistically significant taxa (Table 3), and intersection of the results of the three analysis indicated which taxa may participate in colonization resistance to *C. difficile*. We found that the major number of taxa belonged to the order Clostridiales (Firmicutes), specifically to the families Ruminococcaceae (*Ruminococcus, Subdoligranulum*, and *Gemmiger*), Oscillibacteraceae (*Oscillibacter*) and Eubacteriaceae (*Anaerovorax*). We also found unclassified Ruminococcaceae and Erysipelotrichaceae belonging to the Clostridiales and Erysipelotrichales orders, respectively, as well as other Clostridia and Clostridiales members. Finally, the genus *Escherichia* from the family Enterobacteriaceae, Proteobacteria phylum, was also detected.

**Table 3:** **Significant taxa and associated *p*-value resulting from the three comparative analyses to find protective candidate taxa**.

**Taxa**	**Comparison**	**Comparison**	**Comparison**
	**1(a)**	**2(b)**	**3(c)**
*Akkermansia*	0	1.66E-4	NS
*Anaerotruncus*	4.62E-6	NS	NS
***Anaerovorax***	1.12E-70	1.43E-3	3.41E-3
Clostridium cluster IV	6.11E-14	NS	1.27E-32
Clostridium cluster XlVb	0.02	NS	NS
Clostridium cluster XVIII	2.16E-18	6.44E-73	NS
*Enterococcus*	2.63E-7	2.62E-258	NS
Erysipelotrichaceae incertae sedis	1.46E-6	1.23E-6	NS
***Escherichia***	0	6.48E-22	6.01E-12
*Faecalibacterium*	6.71E-5	NS	NS
***Gemmiger***	0.07	0.05	3.41E-3
*Holdemania*	0.07	1.52E-11	NS
***Oscillibacter***	0	1.33E-15	8.23E-42
*Pseudoflavonifractor*	5.42E-16	NS	NS
*Pyramidobacter*	0.04	NS	NS
***Ruminococcus***	4.85E-228	1.19E-6	5.25E-10
***Subdoligranulum***	1.03E-19	2.76E-8	3.41E-3
**uc_Clostridia**	1.62E-3	4.27E-5	3.46E-5
**uc_Clostridiales**	2.35E-11	0.06	1.57E-18
Clostridiales incertae sedis XIII	1.62E-3	0.04	NS
uc_Enterobacteriaceae	3.18E-6	1.73E-17	NS
**uc_Erysipelotrichaceae**	0.02	0.07	3.46E-5
**uc_Ruminococcaceae**	0	7.85E-117	4.44E-129
*Anaeroglobus*	NS	NS	1.47E-33
*Bacteroides*	NS	NS	5.85E-11
*Dialister*	NS	NS	5.25E-10
*Selenomonas*	NS	NS	4.73E-9
uc_Betaproteobacteria	NS	NS	0.04
uc_Lachnospiraceae	NS	NS	1.50E-9

In bold are the candidate taxa that were significant in the three comparisons. (a) (F_before and H_before) vs. (F_16D, H35_D, and H38_D). (b) A, B, C, D, and E samples before vs. during AB treatment. (c) (H35_D and H38_D) vs. (H_after).

**Figure 4:**
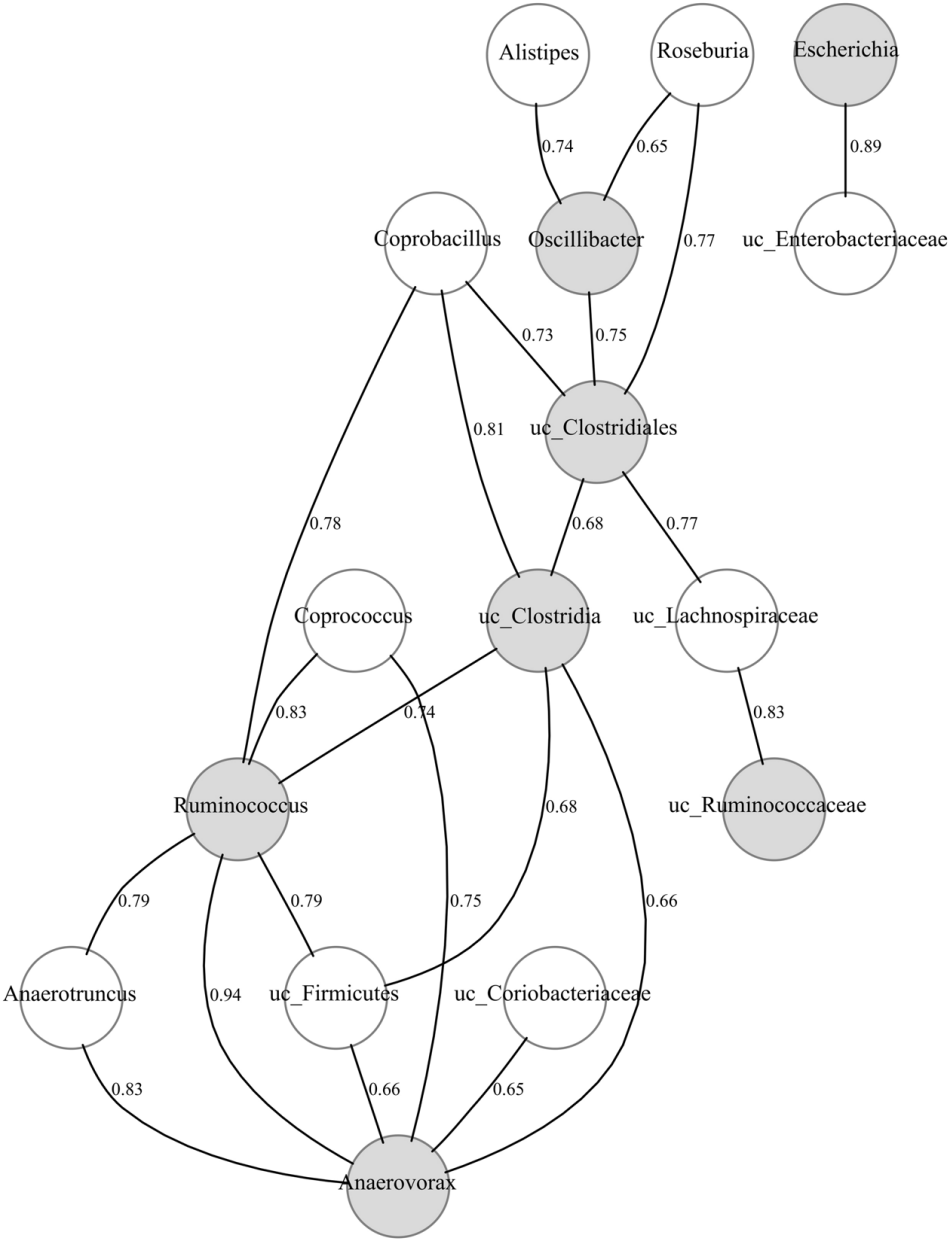
**Bayesian network of microbial composition in CD− samples of patients (A–E), during treatment, and the candidate protective taxa.** The significant positive correlations (*p*-value < 0.01) between the candidate protective taxa and other members of the bacterial community are shown. The correlation coefficients are indicated. Gray and white nodes represent candidate and correlated taxa, respectively. Candidate taxa with no-correlations are not included.

Once the candidate protective taxa had been detected, we performed a Bayesian network (see Materials and Methods) to find other related members of the bacterial community and hence also putatively involved in pathogen colonization resistance (Figure 4). *Gemmiger, Subdoligranulum* and uc_ Erysipelotrichaceae did not show any significant correlation and thus they are not represented in the figure. It is worth noting that most taxa showing a positive and significant correlation with the candidates were phylogenetically related to them, mainly belonging to the Clostridiales order, such as *Roseburia* and *Coprococcus* (Lachnospiraceae family) and *Anaerotroncus* (Ruminococcaceae family).

### Functional changes in patients developing CDI

In the present work, we performed the functional annotation of the 12 metagenomes sequenced (metagenome of sample F_after could not be analyzed, see Materials and Methods) by comparison against the TIGRFAM database, obtaining the following hierarchical classification: main roles (the highest functional level), sub roles (more specific metabolic functions for each one of the main roles) and genes (metabolic functions) for all the reads. Figure S2 shows great homogeneity in the main role distribution of different samples for all three patients (F, G, and H). On average, the most abundant main role categories were: energy metabolism (12.3% ± 2.1), protein synthesis (12% ± 2), transport and binding proteins (8.6 ± 2.5%) and cell processes (8.6 ± 1.3%). Similar main role distribution was described for patients A, B, C, and D in our previous study (Pérez-Cobas et al., 2013b), which is expected due to the importance of these household functions for the survival and growth of gut bacteria.

In patient F, there were 51 significantly different sub roles between samples corresponding to before and during AB treatment (F_before vs. F16_D) (Table S3). The most significant over-represented categories in AB treated samples were: DNA metabolism/chromosome-associated proteins; cellular processes/DNA transformation; cell envelope/biosynthesis and degradation of surface polysaccharides and lipopolysaccharides; and energy metabolism/ pentose phosphate pathway. The under-represented categories were: protein synthesis/tRNA aminoacylation; transport and binding proteins/amino acids; peptides and amines; and cell envelope/surface structures.

Three different comparisons were made for patient H: (i) before vs. during treatment but before *C. difficile* detection (H_ before vs. H7_D, H14_D and H20_D); (ii) before vs. CD+ samples (H_before vs. H35_D and H38_D) and (iii) CD− prior to CDI vs. CD+ samples (H14_D and H20_D vs. H35_D and H38_D). In total, we found 37 significant sub roles increased or decreased in CD− samples. (i) Those that increased during AB but before CDI were mainly involved in “cell processes/DNA transformation” and “protein synthesis/translation factors,” whereas we observed a significant decrease in “amino acid biosynthesis/folic acid”; “mobile and extrachromosomal elements function/plasmid functions”; “signal transduction/PTS” and “transport and binding proteins/carbohydrates, organic alcohol and acids.” (ii). A similar functional profile was found when we compared before vs. CD+ samples. (iii) Finally, when we specifically compared the two samples previous to infection with the two CD+ samples we found 54 significant sub-roles. The most significant over-represented in the CD+ samples were: “signal transduction/PTS”; “transport and binding proteins/carbohydrates, organic alcohols, and acids”; “transport and binding proteins/amino acids, peptides and amines” and “cell envelope/biosynthesis and degradation of mureinsacculus and peptidoglycan” (Table S3).

Patient G proved to be infected by the pathogen throughout the study. Thus, we compared the sub roles distribution, before vs. during treatment, to find those functions that could be AB-related. The comparison showed that only two categories changed during AB: “amino acid biosynthesis/serine family” decreased (*p* = 0) while “cell envelope/other” increased (*p* = 0.04).

### Differences in the functional profile between *c. difficile*-infected and non-infected patients

To compare the whole functional composition of CD+ sample of patients F, G, and H with CD− samples during treatment of patients A, B, C, D, and E, we applied a correspondence analysis based on TIGRFAM functions abundance, with both axes explaining a total of 49.3% of sample variance. The analysis did not show a clear differential functional pattern between the CD+ and CD− groups given the CD+ samples seem to be a subset of the CD− group (Figure 3B). We also used the Adonis test to evaluate the significance of ABs in structuring the functional profile of the microbial community in a different way for the two groups (CD+ and CD−). The factor was not significant at the hierarchical level sub roles and metabolic functions, the *p*-values being 0.63 and 0.73, respectively. To find specific sub roles that could be associated to CD+ samples, we compared the functional profile of the same previously tested samples (Table 4), finding significant enrichment in “transport and binding proteins,” mainly for “carbohydrates, organic alcohols and acids,” and “signal transduction” by the phosphotransferase system (PTS). However, “mobile and extrachromosomal element functions” and “aromatic amino acid family biosynthesis” were significantly underrepresented.

**Table 4:** **Comparisons of sub-roles distribution between CD− (during time points of A–E patients) and CD+ (F16_D, F_after, G_before, G4_D, G_after, H35_D and H38_D) samples**.

**Main role**	**Sub-role**	***P*-value**
Amino acid biosynthesis	Aromatic amino acid family	↓1.5E-29
Biosynthesis of cofactors, prosthetic groups, and carriers	Pantothenate and coenzyme A	↓8.2E-7
	Riboflavin, FMN, and FAD	↑1.2E-2
Cellular processes	DNA transformation	↓1.0E-3
	Sporulation and germination	↓1.2E-2
	Toxin production and resistance	↓4.9E-3
Central intermediary metabolism	Amino sugars	↑2.0E-6
	One-carbon metabolism	↑2.3E-2
	Other	↑3.9E-6
	Phosphorus compounds	↓6.0E-4
	Sulfur metabolism	↓8.6E-3
DNA metabolism	Chromosome-associated proteins	↑6.1E-8
	Degradation of DNA	↓1.4E-2
	DNA replication, recombination, and repair	↑1.1E-2
	Restriction/modification	↓1.0E-3
Energy metabolism	Electron transport	↓1.7E-3
	Fermentation	↑3.0E-4
	Glycolysis/gluconeogenesis	↑1.6E-4
	Pentose phosphate pathway	↑1.5E-2
	Sugars	↑2.3E-2
Hypothetical proteins	Conserved	↓9.4E-7
Mobile and extrachromosomal element functions	Other	↓3.4E-17
Protein synthesis	Other	↑4.7E-2
	tRNA and rRNA base modification	↑8.2E-3
Purines, pyrimidines, nucleosides, and nucleotides	2′-Deoxyribonucleotide metabolism	↓1.4E-2
	Purine ribonucleotide biosynthesis	↑3.6E-7
	Salvage of nucleosides and nucleotides	↑3.0E-4
Regulatory functions	Protein interactions	↓3.6E-5
Signal transduction	PTS	↑1.5E-20
Transcription	Degradation of RNA	↑1.5E-3
	DNA-dependent RNA polymerase	↑1.5E-2
	RNA processing	↑8.2E-3
Transport and binding proteins	Anions	↑9.5E-4
	Carbohydrates, organic alcohols, and acids	↑6.3E-18
	Cations and iron carrying compounds	↑1.2E-5
	Unknown substrate	↑3.6E-5
Unknown function	General	↑2.0E-6

Arrows indicate the sub-roles significantly over-represented (upward) and under-represented (downward) in the CD+ samples.

### Candidate functions involved in *c. difficile* colonization resistance

Just as in the 16S rRNA gene survey, we performed three comparative analyses to find (in the intersection) those metabolic functions that may play a role in colonization resistance. Table 5 shows the roles, sub roles, and functions that may be protective. Those with a clearly assigned role are involved in “aromatic amino acid biosynthesis (chorismate mutase),” “endospore formation (stage IV sporulation protein B and anti-sigma F factor),” “metabolism of amino groups (agmatine diminase),” and “stress response mechanisms (rrf2 family protein, redox-active disulfide protein 2 and glutamate decarboxylase).” Doubled CXXCH domain belongs to a protein of unknown function but it is postulated to be part of c-type cytochromes that participate in electron transfer. UDP-N-acetylglucosamine 4,6-dehydratase participates in the biosynthesis of pseudaminic acid. No sub-roles were assigned to indolepyruvate ferredoxin oxidoreductase and RNA polymerase sigma-70 factor.

**Table 5:** **Candidate functions involved in *C. difficile* colonization resistance**.

**Main role**	**Sub-role**	**Function**
Amino acid biosynthesis	Aromatic amino acid family	Chorismate mutase
Cellular processes	Sporulation and germination	Stage IV sporulation protein B
Regulatory functions	Protein interactions	Anti-sigma F factor
Central intermediary metabolism	Polyamine biosynthesis	Agmatine deiminase
Unknown function	General	rrf2 family protein
		Redox-active disulfide protein 2
NA	NA	Glutamate decarboxylase
		Doubled CXXCH domain
		Indolepyruvate ferredoxin oxidoreductase
		RNA polymerase sigma-70 factor*
		UDP-N-acetylglucosamine 4,6-dehydratase

^*^RNA polymerase sigma-70 factor, Bacteroides expansion family 1.

**Figure 5:**
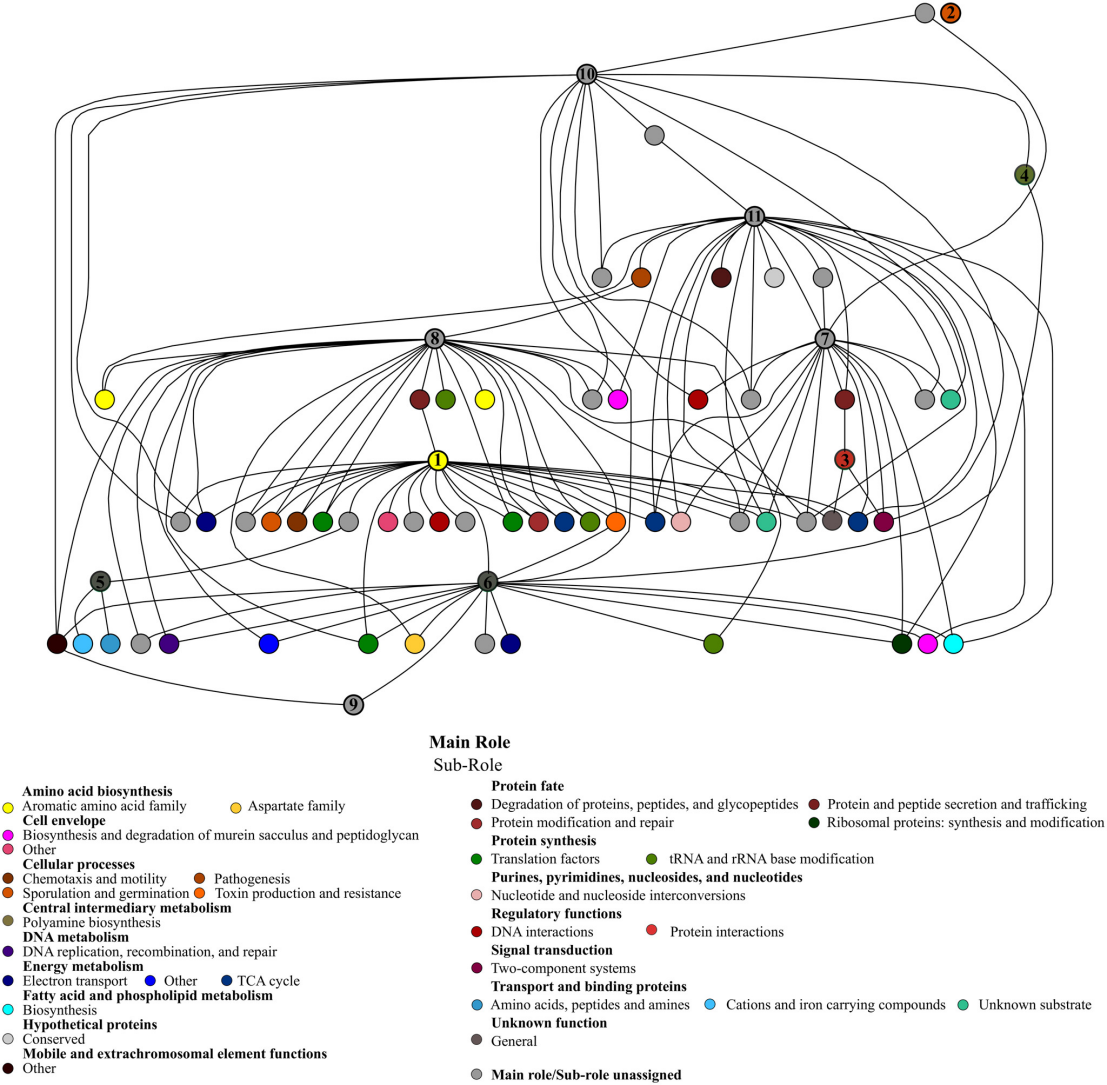
**Bayesian network of microbiota potential functions in CD− samples of patients (A–E), during treatment, and the protective candidate taxa.** The significant positive correlations (*p*-value < 0.01) between the candidate protective functions and other functions of the gut ecosystem are shown. Each node represents a specific function with the corresponding subrole color. The candidate protective function nodes are indicated by numbers: (1) Chorismate mutase; (2) Stage IV sporulation protein B; (3) Anti-sigma F factor; (4) Agmatine deiminase; (5) rrf2 family protein; (6) Redox-active disulfide protein 2; (7) Glutamate decarboxylase; (8) Doubled CXXCH domain; (9) Indolepyruvate ferredoxin oxidoreductase; (10) RNA polymerase sigma-70 factor, Bacteroides expansion family 1; (11) UDP-N-acetylglucosamine 4,6-dehydratase.

We also performed a Bayesian network to find significant and positive associations between the candidate protective functions and other functions that may be important in pathogen infection resistance. Figure 5 shows the functional network according to hierarchical categories. In a general overview, most of the candidate functions were connected with several different sub roles, and correlations between candidates were also found. The most frequently connected function was the doubled CXXCH domain (26 correlations), and chorismate mutase (25 correlations). Additionally, these two candidate functions shared some connections whose nodes are involved in different roles, the majority being related to energy metabolism, protein synthesis and fate, as well as amino acid biosynthesis. The UDP-N-acetylglucosamine 4,6-dehydratase showed 21 correlations, mainly with cell envelope, protein fate and transport system roles. Also, this function was connected to another important candidate: glutamate decarboxylase, with which it shares some correlations. The redox-active disulfide protein 2 and glutamate decarboxylase presented 17 correlations each. The former, which is correlated to the two candidates known as chorismate mutase and indolepyruvate ferredoxin oxidoreductase, showed associations with energy metabolism and protein synthesis, while glutamate decarboxylase is correlated to protein fate, regulatory and transport functions.

## Discussion

In this study, we have analyzed changes in the bacterial composition and functional profile of the gut microbiota of two patients (F and H) that were positive for *C. difficile* (CD+ samples) after AB treatment and one patient (G) that despite not having taken AB was already CD+ when entered to the hospital. Patients F and H had an unusual microbiota at the start of the study (before AB treatment), enriched in *Akkermansia* genus (30.6%) and highly abundant in *Escherichia* genus (85.7%), respectively. We also compared the gut microbiota of those three patients with five individuals from two previous studies (Pérez-Cobas et al., 2013a,b), who were also treated with AB but did not develop CDI. All the patients fit the same inclusion criteria. Despite the heterogeneity of the samples and only 15 time points are overall compared, we consider that the results obtained with the different analyses performed, provide new insights into the effect of CDI on the structure and metabolic functions of the human gut microbiota. Furthermore, we identified members of the bacterial community and metabolic functions that are differentially present in the CD− samples compared to the CD+ samples and thus could be involved in resistance to *C. difficile* colonization.

The gut microbiota of the three CDI patients showed large variations in bacterial composition and diversity throughout the therapy, confirming that antibiotics disturb the ecological equilibrium of microbial communities. Previous studies showed great fluctuations and low diversity of the human gut microbiota under the effects of a wide variety of ABs, although patients did not develop CDI (Dethlefsen et al., 2008; Dethlefsen and Relman, 2010; Pérez-Cobas et al., 2013a,b). In addition to the influence of AB on the microbiota structure, this survey found that CDI contribute to decreasing bacterial diversity since the infected samples showed, in general, lowest biodiversity index values and richness estimators than non-infected samples. In this respect, a mouse colitis model-based study has suggested that intestinal inflammation during colonization by some pathogens, including *C. difficile*, affect microbiota equilibrium (reviewed in Stecher and Hardt, 2011), contributing to reduced microbial diversity.

Similarly, significant alterations in the abundance of some taxa (mainly from the Firmicutes phylum) and a decrease in microbial diversity and species richness were found in individuals with CDI (Antharam et al., 2013).

We have found that the microbiota of the infected samples (CD+) share some common features, being depleted in commensal genera such as *Ruminococcus, Roseburia, Subdoligranulum, Blautia* or *Coprococcus* and enriched in *Lactobacillus, Enterococcus*, Clostridium clusters XlVa and XI. The latter being the phylogenetic cluster which contains the *C. difficile* species (Collins et al., 1994). Although the relative abundance of cluster XI was variable between the infected samples, its presence is higher in CD+ than in CD− samples, probably due to the high abundance of *C. difficile*. The higher abundance of Clostridium cluster XIVa could be a consequence of the microbiota imbalance, since members of this group have been characterized as opportunists (Lozupone et al., 2012). This may also be the case of *Enterococcus*, which is a common opportunistic pathogen that becomes dominant when the normal gut microbiota is disturbed by ABs (Donskey, 2004; Ubeda et al., 2010). *Enterococcus* was also over-represented in samples of reduced biodiversity in other CDI studies (Antharam et al., 2013; Vincent et al., 2013). The higher abundance of *Lactobacillus* in the CD+ samples is also interesting. For example, a murine model-based study found that Lactobacillaceae was dominant in CDI samples (Rea et al., 2011) as did a study of CDI in humans (Antharam et al., 2013). Although *Lactobacillus* has been described as an intestinal probiotic genus, different studies show that only specific strains (e.g., *L. delbrueckii*) can inhibit *C. difficile* growth (Naaber et al., 2004; Banerjee et al., 2009). Further research would be needed to clarify the role of *Lactobacillus* strains in gut colonization by *C. difficile*.

The three comparisons performed enabled us to identify taxa that were significantly over-represented in CD− samples, due to AB therapy, in individuals that either did not develop CDI (comparison 2) or recover from CDI (comparison 3), but decreased in those CD+ samples (Comparison 1). Thus, *Anaerovorax, Escherichia, Gemmiger, Oscillibacter, Ruminococcus, Subdoligranulum*, uc_Clostridia, uc_Clostridiales, uc_Erysipelotrichaceae, and uc_Ruminococcaceae were found as candidates for protecting against *C. difficile* colonization. Bayesian correlation networks are a powerful tool to search and study ecological or metabolic associations in microbial communities (Durbán et al., 2012), and thus we used them to look for other taxa associated to the above, which may be also indirectly involved in resistance by ecologically interacting with the candidates. Most of the taxa in the network belonged to Clostridia: *Ruminococcus, Subdoligranulum, Oscillibacter, Anaerovorax, Roseburia, Coprococcus, Anaerotroncus, Gemminger* and other unclassified members of Lachnospiraceae and Ruminococcaceae families. It has been proposed that competition of normal gut microbiota members with their related pathogens for limiting resources or sites, called “niche exclusion,” could be a colonization resistance mechanism (reviewed in Britton and Young, 2012). Thus, this niche hypothesis could explain the role of these related taxa belonging to Clostridiales in protecting against CDI. In this regard, some studies in mice have shown that Clostridia members, such as Lachnospiraceae, are *C. difficile* antagonists and restore the microbiota when fed to infected mice (Itoh et al., 1987; Reeves et al., 2011, 2012; Lawley et al., 2012). Another study in hamsters showed that non-toxigenic *C. difficile* were able to prevent the toxigenic pathogen (Sambol et al., 2002; Merrigan et al., 2003), suggesting a more efficient utilization of limiting nutrients (niche exclusion) as the protection mechanism. In human studies, members of the Ruminococaceae and Lachnospiraceae families were significantly depleted in CDI patients (Antharam et al., 2013).

Some of the Clostridia members found to be associated to the main protective candidate taxa, such as *Roseburia* or *Coprococcus*, are active anaerobic short-chain fatty acids (SCFA) producers (Barcenilla et al., 2000; Pryde et al., 2002). This could be other mechanism through they are candidates to protect against CDI, since SCFA are reported to inhibit *C. difficile* growth and also to decrease the production of toxin *in vitro* (May et al., 1994). Moreover, it has been postulated that the anaerobic fraction of the microbiota is essential for gut ecosystem stability in healthy individuals, because the butyrate and other SCFAs they produce have anti-inflammatory effects and stimulate the immune system and, thus, this imbalance increases the risk of *C. difficile* overgrowth (Bartlett, 2002; Roy et al., 2006; Jernberg et al., 2010). However, a recent study in mice found that SCFA production was no correlated with lower levels of *C. difficile* colonization (Reeves et al., 2012). In addition, these authors found that the microbiota composition of CDI mice was partially restored when they used only one isolate of the Lachnospiraceae family for inoculation. Nevertheless, total restoration was obtained when total fecal content was transferred from a wild-type mouse. These results agree with our findings because we have found several putative candidate protective taxa, indicating that more than one bacterial group is involved in pathogen protection. Hence, further research should test *in vivo* the colonization resistance capacity of the specific ensemble we have proposed.

In a previous study, we showed that the metabolic profiles of AB-associated shifts in human gut microbiota were less dramatic than those in bacterial composition, principally when considering main roles. This is due to functional redundancy of the human gut microbiota, and the fact it has a very general set of functions (Pérez-Cobas et al., 2013b). We have also found great homogeneity in distribution of the main role in all the samples. However, differences appear when considering more inclusive functional levels (sub-roles and functions). In this study, patients showed different functional responses (sub-roles) to ABs, in agreement with our previous study where a great inter-individual variability was found in AB-treated patients. Although no significant differences between both groups of AB-treated patients (CDI and non-infected) as a whole were detected, a specific functional profile was found. Thus, the transport, metabolism, and regulation of sugars such as mannose, fructose, lactose, glucitol, or mannitol were over-represented functions in CDI samples, the major sugar transport system being the phosphotransferase system (PTS). In a previous work, we found that AB increases PTS in metagenomes, since it seems to give advantage to bacteria carrying them under stress conditions (Deutscher et al., 2006; Pérez-Cobas et al., 2013b). The higher presence of these functions in CD+ samples compared to CD− is noteworthy, even when both were treated with ABs, because it could be related with the infection, as shown in a metabolomic study in mice that developed CDI (Theriot et al., 2014). The same authors found an increase in carbohydrates like mannose, fructose, lactose, glucitol, or mannitol after AB treatment, and they postulated that these increases favored *C. difficile* germination and growth. Related to this finding, a transcriptomic study revealed that sugars released by an altered microbiota are exploited by enteric pathogens such as *Salmonella enterica* and *C. difficile* (Ng et al., 2013). Thus, *C. difficile* and other opportunistic bacteria can efficiently catabolize the excess of carbohydrates generated by the disrupted microbiota and, in the absence of competitors, increase colonization rates.

Using the same three comparisons, we also found metabolic functions that may play a role in *C. difficile* colonization resistance (Table 5). Overall, there was a higher abundance of functions related to aromatic amino acid biosynthesis, being chorismate mutase the central node of the network, since it was strongly connected to other important functions like energy metabolism or protein fate. The chorismate mutase, which participates in tyrosine, phenylalanine and tryptophan biosynthesis, could be involved in colonization resistance through stimulation of the immune system, since the tryptophan metabolite participates in immune system equilibrium and inflammation regulation (Zelante et al., 2013). Future research should be conducted to discover the mechanism by which aromatic amino acid synthesis could protect against colonization by *C. difficile*. Also, some energy metabolism pathways seem important, such as TCA cycle, electron transport, or fatty acid biosynthesis. A great number of different transporter families, regulator genes, and genes involved in responses to osmotic or acid stress were also highlighted in the network, possibly playing a role in colonization resistance.

Another possible protective pathway was peptide catabolism via tryptophan metabolism. Low abundance of protein digestion markers was associated to susceptibility to CDI in the mouse gut (Theriot et al., 2014). Regarding host immune response, we found polyamine biosynthesis (putrescine or cadaverine) by decarboxylation of amino acids to be another potential protective pathway. A previous study reported that these metabolites interact with the gut microbiota, stimulating the immune system and playing a role in intestinal maturation (Gómez-Gallego et al., 2012). In this regard, Jung et al. (2003) found that glutamate decarboxylase activity, related to polyamines, was also a protective determinant, playing a role in protection against acid stress. It is also relevant that this enzyme is connected to other functions in the network, such as protein fate, transcription regulation, or transport systems, thus reinforcing its protective role. Moreover, other protective gene-products regulate metabolic pathways that are important for several cellular physiology processes, like osmotic stress resistance and responses to environmental changes (Wouters et al., 2010; Shepard et al., 2011).

In summary, we found specific fecal microbiota in CDI patients as it was enriched in *Lactobacillus, Enterococcus*, Clostridium clusters XIVa and XI but depleted in SCFA-producing bacteria. The latter bacterial group could be involved in *C. difficile* colonization resistance. A group of metabolic processes related to the metabolism of proteins, amino acids and responses to stress would seem to participate in avoiding pathogen invasion in the human gut ecosystem. Further research into these pathways should be undertaken to unravel the mechanism by which they participate in colonization resistance to *C. difficile*. A larger cohort of patients with similar sampling would be needed to deeper define the CDI microbiota at taxonomic and functional level.

## Author contributions

Andrés Moya, María J. Gosalbes, and Amparo Latorre conceived the work. Ana E. Pérez-Cobas performed all the analyses. María J. Gosalbes and Alejandro Artacho help with some of the analyses. The manuscript was written by Ana E. Pérez-Cobas, María J. Gosalbes, and Amparo Latorre. Andrés Moya and Stephan J. Ott revised the manuscript.

### Conflict of interest statement

The authors declare that the research was conducted in the absence of any commercial or financial relationships that could be construed as a potential conflict of interest.
