# Supplementary material for: Structural and functional changes in the gut microbiota associated to Clostridium difficile infection
Source: Front Microbiol. 2014 Jul 4;5:335. doi: 10.3389/fmicb.2014.00335 (PMC4163665; doi:10.3389/fmicb.2014.00335)
Supplement: Supplementary file 1 [file DataSheet1.PDF]

Supplementary Material

Structural and functional changes of the gut microbiota associated to *Clostridium difficile* infection

Ana Elena Pérez-Cobas[1,2], Alejandro Artacho[1], Stephan J. Ott[3,4], Andrés Moya[1,2], María José Gosalbes[1,2] and Amparo Latorre[1,2*].*

[1]Unidad Mixta de Investigación en Genómica y Salud de la Fundación para el Fomento de la Investigación Sanitaria y Biomédica de la Comunidad Valenciana (FISABIO) y el Instituto Cavanilles de Biodiversidad y Biología Evolutiva de la Universitat de València, Valencia, Spain.

[2]CIBER en Epidemiología y Salud Pública (CIBERESP), Madrid, Spain.

[3]Institute for Clinical Molecular Biology at the Christian-Albrechts University, Kiel, Germany.

[4]Department for Internal Medicine, University Hospital Schleswig-Holstein, Campus Kiel, Kiel, Germany.

[*]These authors contributed equally to this paper.

Correspondence:
Dr. Amparo Latorre
Instituto Cavanilles de Diversidad y Biológica Evolutiva
Genética Evolutiva
C/ Catedrático José Beltrán, 2
46980, Paterna, Valencia, Spain

SUPPLEMENTARY TABLES AND FIGURES

Supplementary Table 1. Biodiversity measures for *C. difficile* positive patients F, G and H based on OTUs (97%).

Sample	CD (+/-)	N	Shannon	Chao1	SE (Chao1)	ACE	SE (ACE)
F_before	CD-	211	2.83	255.2	16.99	255.54	7.99
F16_D	CD+	114	2.48	172.58	30.18	158.46	6.34
F_after	CD+	31	2.38	49	49.09	44.63	3.12
G_before	CD+	272	4.31	311.84	15.39	309.99	8.63
G4_D	CD+	357	4.77	433.78	22.06	428.53	10.11
G_after	CD+	158	2.45	236.55	26.97	263.6	9.98
H_before	CD-	45	2.44	52.2	9.02	51.68	3.51
H7_D	CD-	79	3.39	95.87	10.32	107.61	5.68
H14_D	CD-	102	3.58	123	14.34	125	5.5
H20_D	CD-	187	3.72	216.4	11.51	229.16	7.52
H35_D	CD+	53	2.25	87	36.98	72.82	4.45
H38_D	CD+	114	2.85	177.91	33.8	155.8	6.43
H_after	CD-	112	4.09	140.96	13.41	147.94	6.32

N: Number of OTUs; SE (Standard Error); ACE: Abundance coverage estimator; NaN: not a number.

Supplementary Table 2. Differential taxa abundance between CD+ (H35_D, H38_D) and CD- samples (H14_D, H20_D) in patient H.

Bacterial taxa	Abundance in CD+ samples	P-value
Lactobacillus	increase	0
Streptococcus	increase	1.49E-47
uc_Lactobacillaceae	increase	9.24E-10
Proteus	increase	3.83E-4
Sutterella	increase	3.74E-3
Bacteroides	decrease	7.33E-301
Escherichia	decrease	6.73E-165
Klebsiella	decrease	1.18E-145
Enterococcus	decrease	6.43E-10
Raoultella	decrease	3.72E-3
Clostridium cluster XlVa	decrease	1.31E-2

Supplementary Table 3. Comparisons of the sub-role abundance and p-value associated. Patient F: (B/D+) before and during therapy (F_before *vs* F16_D). Patient H: (B/D-) before *vs* during the treatment but prior *C. difficile* detection (H_before *vs* H7_D, H14_D and H_20_D); (B/D+) before *vs* CD+ samples (H_before *vs* H35_D and H38_D); (D-/D+) during AB (H14_D and H20_D *vs* H35_D and H38_D). Arrows show sub-roles more (upward) and less (downward) abundant. NS, not significant.

		Patient F	Patient H		
Main Role	Sub-Role	B/D+	B/D-	B/D+	D-/D+
Amino acid biosynthesis	Aromatic amino acid family	NS	↑0.02	NS	↓3E-4
	Aspartate family	NS	↑0.02	↑2E-3	NS
	Glutamate family	↓0.01	↓0.02	NS	↑5E-5
	Histidine family	NS	NS	↓2E-3	↓2E-4
	Pyruvate family	NS	↓0.04	↓2E-4	NS
	Serine family	NS	NS	NS	↓0.02
Biosynthesis of cofactors, prosthetic groups, and carriers	Biotin	↑4E-3	NS	↓3E-3	↓3E-5
	Chlorophyll and bacteriochlorphyll	↓0.04	NS	NS	NS
	Folic acid	NS	↓9E-14	↓2E-26	NS
	Heme, porphyrin, and cobalamin	NS	NS	NS	NS
	Menaquinone and ubiquinone	↓0.04	↓0.04	NS	↑0.01
	Molybdopterin	NS	NS	NS	↑0.03
	Other	NS	NS	↓4E-3	↓5E-3
	Pyridine nucleotides	NS	NS	NS	↑0.03
	Pyridoxine	↑4E-3	NS	NS	↓0.01
	Riboflavin, FMN, and FAD	↑0.02	NS	NS	NS
Cell envelope	Murein sacculus and peptidoglycan*	↓2E-6	↓0.03	↑4E-3	↑8E-12
	Surface polysaccharides**	↑7E-19	NS	NS	NS
	Other	↓0.04	NS	NS	NS
	Surface structures	↓2E-11	↓2E-7	↓8E-12	NS
Cellular processes	Adaptations to atypical conditions	↑2E-6	NS	NS	NS
	Biosynthesis of natural products	↓4E-3	NS	NS	↑0.02
	Cell division	↓1E-4	NS	NS	NS
	Chemotaxis and motility	NS	↓0.02	↓3E-2	NS
	Detoxification	↑0.01	NS	NS	NS
	DNA transformation	↑6E-19	↑5E-8	NS	↓2E-7
	Pathogenesis	↓0.01	↓0.01	↓7E-3	NS
	Sporulation and germination	↓0.01	↑0.01	NS	↓5E-3
	Toxin production and resistance	↓0.02	↑2E-4	NS	↓2E-6
Central intermediary metabolism	Amino sugars	NS	↓2E-6	↓4E-5	NS
	Nitrogen fixation	NS	↑4E-3	NS	↓5E-3
	Nitrogen metabolism	NS	NS	↓2E-3	NS
	One-carbon metabolism	NS	NS	↑1E-2	NS
	Other	NS	NS	↑2E-2	↑5E-3
	Phosphorus compounds	↓2E-4	NS	↑5E-2	↑3E-3
	Polyamine biosynthesis	NS	↑0.01	NS	↓3E-4
	Sulfur metabolism	NS	NS	↓4E-4	↓5E-3
DNA metabolism	Chromosome-associated proteins	↑9E-37	↑2E-5	NS	↓2E-7
	Degradation of DNA	↓3E-3	NS	NS	NS
	DNA replication, recombination and repair	↓5E-3	NS	↑2E-3	↑4E-5

	Restriction/modification	NS	NS	↓1E-2	NS
	Aerobic	NS	NS	NS	↑0.01
Energy metabolism	Amino acids and amines	↑2E-5	NS	NS	↓0.01
	ATP-proton motive force interconversion	↑3E-3	NS	↑1E-2	↑0.02
	Biosynthesis/degradation of polysaccharides	↓2E-8	↓0.01	NS	↑2E-6
	Electron transport	↑0.03	NS	↓3E-5	↓3E-10
	Entner-Doudoroff	NS	↓0.01	NS	NS
	Fermentation	↑1E-3	↓0.02	↑2E-2	↑1E-7
	Glycolysis/gluconeogenesis	NS	NS	↑2E-3	↑0.05
	Other	NS	NS	NS	↓0.01
	Pentose phosphate pathway	↑8E-11	↓0.01	NS	NS
	Photosynthesis	↑0.01	↑2E-4	NS	↓0.01
	Pyruvate dehydrogenase	↓2E-5	NS	NS	NS
	Sugars	↓0.01	↓0.01	NS	↑0.01
	TCA cycle	NS	NS	↓2E-3	↓2E-3
Fatty acid+	Biosynthesis	↓0.02	NS	NS	NS
	Degradation	NS	NS	NS	↑0.05
Hypothetical proteins	Conserved	↓0.04	NS	NS	NS
	Domain	↓0.04	NS	NS	NS
Mobile and	Other	NS	NS	↓3E-5	↓2E-11
extrachromosomal	Plasmid functions	NS	↓6E-10	↓4E-25	↓6E-4
element functions	Prophage functions	NS	↑0.05	NS	NS
	Transposon functions	NS	NS	↑3E-2	NS
Protein fate	Proteins, peptides, and glycopeptides***	NS	NS	↑1E-3	↑2E-3
	Protein and peptide secretion and trafficking	↓2E-3	NS	NS	NS
	Protein folding and stabilization	↓2E-4	NS	↑3E-2	↑1E-5
	Protein modification and repair	↓0.04	NS	NS	NS
Protein synthesis	Other	NS	NS	↑8E-3	NS
	Ribosomal proteins: synthesis/modification	NS	↑3E-9	↑4E-6	NS
	Translation factors	NS	↑4E-8	↑1E-3	↓0.03
	tRNA aminoacylation	↓2E-14	NS	↑1E-3	↑5E-7
	tRNA and rRNA base modification	NS	↑0.02	NS	↓9E-6
Purines, pyrimidines,	2'-Deoxyribonucleotide metabolism	NS	NS	↑8E-3	NS
nucleosides, and	Purine ribonucleotide biosynthesis	NS	NS	↑4E-5	↑3E-3
nucleotides	Pyrimidine ribonucleotide biosynthesis	↓0.02	NS	↑2E-3	NS
	Salvage of nucleosides and nucleotides	↑0.02	NS	NS	↑0.02
Regulatory functions	DNA interactions	NS	↓0.04	↓2E-3	NS
	Protein interactions	NS	NS	↓2E-3	NS
	Other	↓3E-6	NS	↑3E-3	↑0.01
	Small molecule interactions	↑0.01	NS	NS	NS
Signal transduction	PTS	NS	↓1E-16	NS	↑5E-16
	Two-component systems	↑0	↑4E-5	NS	↓1E-5
Transcription	DNA-dependent RNA polymerase	NS	↑0.01	↑2E-14	↑3E-7
	RNA processing	↑0.02	↓0.05	NS	NS
	Transcription factors	↓0.04	NS	NS	NS
Transport and	Amino acids, peptides and amines	↓3E-12	↓1E-7	NS	↑3E-12
binding proteins	Anions	↓9E-9	↓7E-8	NS	↑3E-8
	Carbohydrates, organic alcohols and acids	NS	↓10E-35	↓3E-8	↑1E-12
	Cations and iron carrying compounds	NS	↓1E-3	NS	↑0.01

	Nucleosides, purines and pyrimidines	↑3E-3	↓0.02	NS	↑0.03
	Other	NS	↓2E-6	NS	↑4E-8
	Unknown substrate	↑0.03	NS	↑1E-2	NS
Unknown function	Enzymes of unknown specificity	↓2E-10	NS	NS	↑0.04
	General	↑0.04	NS	NS	NS

+Fatty acid and phospholipid metabolism.

*Biosynthesis and degradation of murein sacculus and peptidoglycan.

**Biosynthesis and degradation of surface polysaccharides and lipopolysaccharides.

***Degradation of proteins, peptides, and glycopeptides.

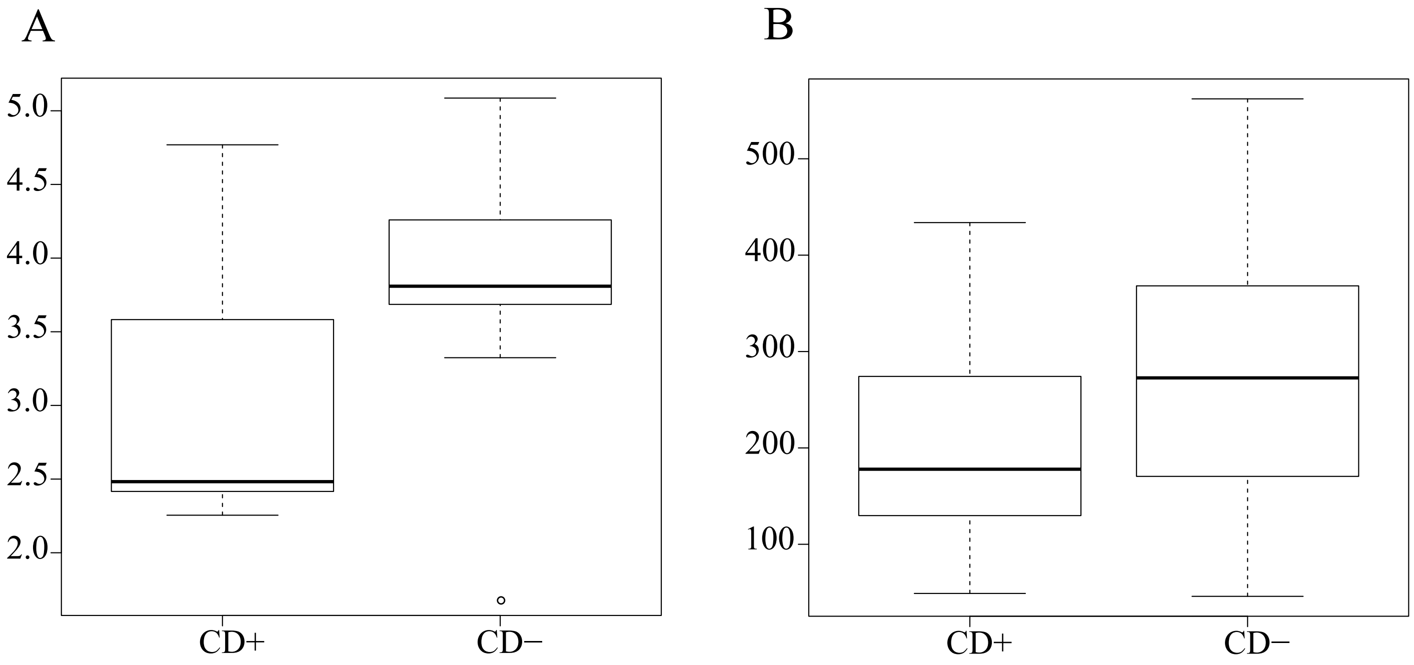

Supplementary Figure 1. Diversity analyses in CD+ (n=7) and CD- (n=15) samples. (A)Shannon index and (B) Chao 1 estimator of CD+ (F_16D, F_after, G_before, G4_D, G_after, H_35D and H_38D) and CD- (patients A, B, C, D and E during AB) samples. Thickest line indicates the median.

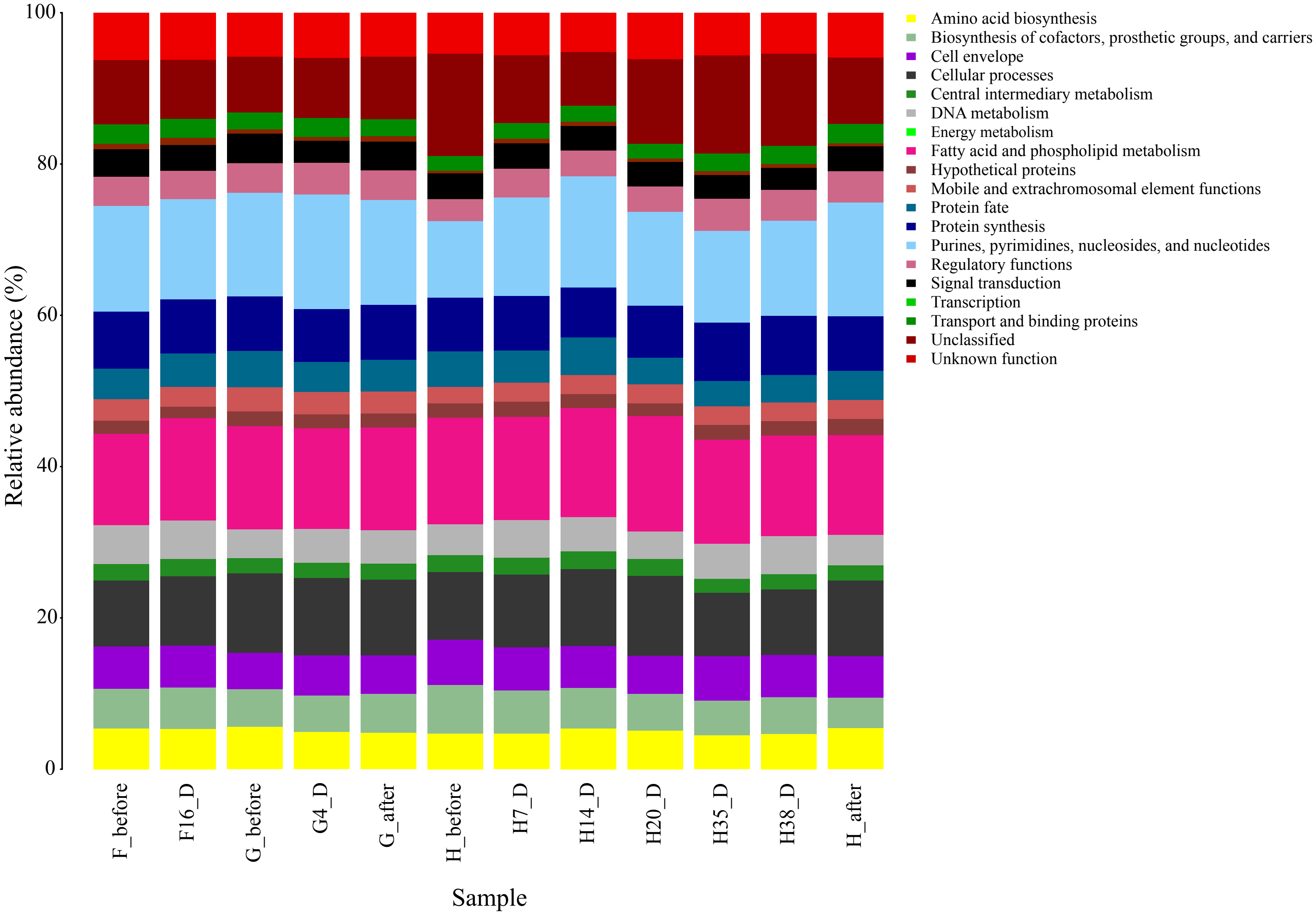

Supplementary Figure 2. Relative abundance of the main functional roles of samples from patients F, G and H.
